# Peace through health: traditional medicine meditation in the prevention of collective stress, violence, and war

**DOI:** 10.3389/fpubh.2024.1380626

**Published:** 2024-04-03

**Authors:** Robert H. Schneider, Michael C. Dillbeck, Gunvant Yeola, Tony Nader

**Affiliations:** ^1^Institute for Prevention Research, Vedic City, IA, United States; ^2^College of Integrative Medicine, Maharishi International University, Fairfield, IA, United States; ^3^Dr. Tony Nader Institute for Consciousness and Its Applied Technologies, Maharishi International University, Fairfield, IA, United States; ^4^Department of Kayachikitsa, Dr. D. Y. Patil College of Ayurveda and Research Center, Dr. D. Y. Patil Vidyapeeth, Pune (Deemed to be University), Pimpri, Pune, India

**Keywords:** war, population health, meditation, Transcendental Meditation, collective stress, collective violence, armed conflict, traditional medicine 2

## Abstract

In the midst of global armed conflicts, notably the Israel-Hamas and Ukraine-Russia wars, there is an urgent need for innovative public health strategies in peacebuilding. The devastating impact of wars, including mortality, injury, disease, and the diversion of healthcare resources, necessitates effective and durable interventions. This perspective aligns with WHO recommendations and examines the role of evidence-based meditation from Ayurveda and Yoga in public health to mitigate collective stress and prevent collective violence and war. It highlights the Transcendental Meditation program, recognized for reducing stress, with contemporary evidence supporting its effectiveness in mental health, mind–body disorders, cardiovascular disease, and public health. Empirical studies with cross-cultural replications indicate that these Traditional Medicine meditation practices can reduce collective stress and prevent collective violence and war activity while improving quality of life. The mechanisms of group meditation in mitigating collective violence are explored through public health models, cognitive neuroscience, population neuroscience, quantum physics principles, and systems medicine. This perspective suggests that Transcendental Meditation and the advanced TM-Sidhi program, as a component of Traditional Medicine, can provide a valuable platform for enhancing societal well-being and peace by addressing brain-based factors fundamental to collective stress and violence.

## Introduction

War and armed conflicts cause severe damage to public health through widespread injuries, diseases, disabilities, premature deaths, displaced populations, environmental contamination, and often violations of human rights and international humanitarian law. Moreover, it redirects crucial resources from health and social services to conflict-related activities, potentially perpetuating further violence (1).

The current Israel-Hamas war is part of an “intractable conflict” that has lasted at least 75 years (2). The persistent and ongoing collective violence in the Middle East bears a significant portion of the worldwide burden of immediate, long-term, and inter-generational impacts on health. Other major ongoing wars include Russia-Ukraine, Sudan, Myanmar, and Maghreb (3). Such intractable conflicts, without apparent end demand innovative approaches to peacebuilding beyond conventional diplomacy, military interventions, and public health (2, 4).

### Preventing collective violence

Amidst escalating tensions that threaten to reverberate beyond regional borders, the imperative for healthcare professionals to engage in public health strategies to mitigate and prevent collective violence is underscored by a growing body of evidence and public outcry from international leaders in medicine (5).

The *Lancet* Commission declared that these and related crises *have exposed the inadequacy of national and global leadership and governance structures* (6). Levy and others emphasize the relationship between peace and health. Peace is vital for nations and their populations to achieve their utmost health potential. Conversely, health is a determinant of peace, as expressed in the *peace through health* approach proposed by Arya and others (7).

### Traditional medicine for prevention of collective violence

The World Health Organization (WHO) upholds that preventing collective violence and war is a critical public health mandate (8, 9). WHO initiatives are rooted in recognizing that societal health and prosperity are fundamentally intertwined with the state of peace (6, 10). Further, recent declarations of WHO and G20 Leaders recognize Traditional, Complementary, and Integrative Medicine (TCIM) for its vital role in preventive medicine and public health (11, 12).

Bridging the seemingly disparate themes of prevention of collective violence and Traditional Medicine aligns with the contemporary vision for public health of peace through health (7). This convergence underscores a paradigm where healthcare is not merely reactive but proactively contributes to peacebuilding and society-building (6, 10).

Using the host-agent-environment triad model of public health, Levy proposes that strategies can be developed to prevent armed conflicts. In this context, we present a model to prevent collective violence based on strategies for improving the health and well-being of the people or host element of the triad rather than the agent/military or environment/external factors (10).

### A traditional medicine meditation program for reduction of individual and collective stress and prevention of collective violence

WHO endorses the incorporation of TCIM, notably Ayurveda and Yoga, from the traditional Vedic medicine of India, acknowledging their contributions not just to individual health concerns but also as crucial resources for preventive medicine and public health (11, 12). In Ayurveda, public health is described in the section on *Janapadodhvansa* (13, 14). This traditional discipline addresses the causes of war and outlines preventive strategies. Ayurveda recommends reducing stress by settling the mind and body and experiencing the innermost self to promote mental, physical, and behavioral health with a meditative practice. These descriptions can be found in *Satvavajaya* (mental health), *Sadvritta* (behavior recommendations for social harmony) and *Achar Rasayana* (behavioral recommendations for health and long life) (13, 15).

One technology from Ayurveda and Yoga, that has been revived and extensively investigated in contemporary settings for individual and collective stress reduction is the Transcendental Meditation technique (16, 17). Transcendental Meditation is described as a simple, natural technique for allowing mental activity to spontaneously settle down and experience a state of inner silence or transcendental consciousness (16, 17). Practitioners report experiences of peace, unity, and transcendence during and after the practice (17). Studies conducted across several continents and cultures indicate that it may be easily learned, practiced, and clinically effective regardless of education, culture, language, religion, or philosophy (17) (See below.)

The literature of Ayurveda and Yoga describes how regular practice of this type of traditional meditation can reduce stress and violent behaviors in society and the individual (1, 11, 12, 15). Over the past 50 years, individual and collective stress reduction hypotheses have been empirically tested for individual health and public health effects, including prevention of collective violence (18).

### On the neuroscience of meditation

*There are many kinds of meditation methods, with different goals and* var*ying techniques,* concluded Nash and Newberg in their updated classification and taxonomy of meditation methods (19). While meditation methods have unique goals and procedural techniques, a shared principle among them is the intention to cultivate a targeted, enhanced mental state (19).

Cognitive neuroscience reviews emphasize that since meditation procedures and goals differ, it is not surprising that empirical studies demonstrate contrasting neurophysiological changes associated with the types of meditation methods (19, 20). Neuroimaging research shows distinct cortical and subcortical activation and deactivation patterns in different types of meditation (19, 20). Based on electroencephalographic (EEG) patterns, Travis and Shear proposed three categories of meditation, providing a neuroscience-based taxonomy that corresponds with phenomenology (20, 21).

The first category involves *focused attention* or concentration on an object of attention. Examples are from Buddhist-derived practices, e.g., Vipassana and Zen. Empirically, these are characterized by the gamma frequency in EEG studies, which is associated with effortful thinking and mental control.

The second category, called *open monitoring* type meditation, prescribes observation of thoughts, emotions, breath or body sensations, observed without judgment. The practice is also described as nonreactive monitoring of the momentary content of experience. Examples are Mindfulness meditations and Zazen, characterized by prominent theta and posterior alpha EEG patterns. These records are associated with attention to internal mental processing.

The third category of meditation is called *automatic self-transcending*. This method involves transcending or “going beyond” usual mental activity, i.e., thoughts, emotions, and bodily awareness. The practice allows the practitioners’ attention to move from active thinking to inner silence without effort. The most commonly studied example is Transcendental Meditation from the Vedic system of Traditional Medicine. The process of transcending is characterized by frontal alpha EEG in the alpha-1 range, which is associated with reduced frontal executive processing. Another characteristic of this category is activating the default mode neural network corresponding to the experience of “relaxed wakefulness” (20).

Further, neuroscientific study of spiritual and religious practices (sometimes called neurotheology) indicates that prayer and meditation practices differ in their methods and neurophysiological correlates (19, 22). Prayer is typically a form of communication involving expressions of gratitude, seeking guidance, or asking for assistance. Meditation, particularly Transcendental Meditation, allows the active thinking mind to settle to a silent inner state or transcendence. Thus, while prayer is typically outwardly directed and communicative, meditation is inwardly focused on experiencing a state of inner silence or relaxed wakefulness (23).

Meta-analyses suggest that during the practice of Transcendental Meditation, the individual experiences a unique neurophysiological state of restful alertness characterized by relatively higher galvanic skin response (GSR) resistance, lower respiration rate, and lower plasma lactate in addition to the neurophysiological changes mentioned above (17, 18).

### Mental and physical health effects of Transcendental Meditation

Individual levels of anxiety and other mental health disorders contribute to public health patterns of mental health. Meta-analyses of controlled clinical studies report that Transcendental Meditation appears distinctively effective in reducing anxiety, especially in participants with high levels of anxiety at baseline and has shown clinical effectiveness in treating posttraumatic stress disorder, burnout, and related symptoms of depression and exhaustion cross-culturally (24–27).

Despite advances in modern medicine and public health, cardiovascular disease (CVD) persists as the leading cause of death and disease globally (28). Moreover, psychosocial and environmental stress increases the risk for CVD (29). These may be considered social determinants of health or public health risk factors. By contrast, a series of randomized controlled trials reported that Transcendental Meditation practice, in conjunction with usual medical care, reduces CVD risk factors, morbidity and mortality (27, 30, 31).

Specifically, meta-analyses and clinical trials show lower rates of hypertension, metabolic syndrome, smoking, and substance abuse; reductions in surrogate markers of atherosclerotic CVD, including carotid artery atherosclerosis and myocardial ischemia; decreased relative risk for mortality, myocardial infarction, and stroke; and reductions in healthcare utilization and costs (27, 30, 31).

### Public health applications

Based on the literature of traditional Vedic medicine (13, 14, 32), Maharishi Mahesh Yogi proposed a theory of collective health which predicted that the practice of traditional meditation techniques could not only reduce stress in the individual but would reduce stress in society at large and favorably impact trends of collective violence (18).

A critical meditation technique in this peace-creating program is the TM-Sidhi program, which is described as an advanced meditation technique to train the individual to sustain thought and activity from the silent state of awareness (transcendental consciousness), thus integrating the experience of pure consciousness with activity and amplifying the effects of meditation in individual and collective functioning. The advanced program is practiced in conjunction with the core Transcendental Meditation technique (17, 18).

It was further predicted that objectively measurable societal effects would occur when the size of a group practicing the Transcendental Meditation and TM-Sidhi program together in one place exceeds the square root of 1 % of the target population (18). This hypothesis has been tested in more than 30 peer-reviewed and published controlled studies (18).

Several of these studies tested the group meditation for peace hypothesis in the Middle East, particularly Israel and Lebanon, in addition to studies in Asia and a recent multi-year time series analysis in the US, as reviewed below.

#### Israel and Lebanon

In a prospective, quasi-experimental research project conducted in Israel, a group of Transcendental Meditation and TM-Sidhi program participants came together in Jerusalem for two months in 1983 (33). The group varied in size throughout this period, depending on individual availability. Improvements were found for measures of war intensity and war deaths in the Israel-Lebanese armed conflict derived by standardized methods of content analysis from daily news sources. Time series cross-correlation analyses indicated that the participant numbers had a leading effect in time on the dependent variables. The study also found significant improvements in indices of quality of life at the city and national levels (33). In response to critiques indicating possible confounding factors, the authors published several re-analyses of the data, which supported their initial findings (34).

A study of group meditation effects in the Lebanon civil war examined seven occasions when there were short-term groups of Transcendental Meditation and TM-Sidhi program participants over a 2.25-year period from 1983 to 1985 in Lebanon, which were large enough for the hypothesized effect on the ongoing conflict. Using a daily database created from nine international and regional news sources by an independent Lebanese rater blind to these hypotheses, Box-Tiao impact analysis indicated reduced conflict intensity, conflict fatalities, and increased cooperation by factions during the seven assemblies of Transcendental Meditation and TM-Sidhi program participants in contrast to all other days. The analysis controlled for seasonality and trends in the data, temperature, holidays, and weekends (35).

#### India, Philippines, Puerto Rico

Box-Tiao ARIMA impact-assessment studies at the state, or province, level examined the impact of groups of TM-Sidhi program participants that came together temporarily on special courses. These studies reported crime reductions in the Union Territory of Delhi, India, a reduction of daily Indian Penal Code totals, in Metro Manila, a reduction of weekly crime index totals, equivalent to the FBI Uniform Crime Index in the U.S. and in Puerto Rico a reduction of monthly Type 1 crimes, comparable to the FBI uniform crime index (36).

#### United States

A recent study on the effects of group practice of the Transcendental Meditation and TM-Sidhi programs on collective violence over 17 years in the United States stands out for its methodological rigor. It employed interrupted time-series analysis and used multiple control variables and statistical tests to ensure the robustness of the results (18). The study found that when a group practicing Transcendental Meditation and the TM-Sidhi program reached the predefined threshold of the square root 1% of the US population, there were significant reductions in indicators of national violence, including homicides, rape, aggravated assault, robbery, drug-related deaths, motor vehicle fatalities and injury fatalities alone and in a composite index. After the group numbers substantially decreased, the public health indicators reverted to their baseline state (18).

This collection of studies accounted for alternative explanations for the observed effects, such as changes in economic conditions, shifts in political leadership, and other secular trends (18).

### Comparison with other methods

While considering meditation methods for reducing collective stress and preventing collective violence and war, it is relevant to compare with intercessory prayer practices and outcomes (37, 38). Above, we outlined fundamental differences in practice between meditation and prayer. Meditation, particularly the transcending types, aims to automatically reduce mental activity and gain a unique neurophysiological state of restful alertness. In contrast, prayer typically involves outward communication, seeking guidance, or requesting assistance, which engages more active neurocognitive pathways (22).

These distinctions have practical implications for efficacy in public health interventions. The empirical evidence for meditation, especially in group settings, suggests a capacity to mitigate collective stress and violence in the population (reviewed above). Conversely, the effectiveness of intercessory prayer in similar contexts is undetermined. A Cochrane systematic review analyzed health outcomes of intercessory prayer for specific individuals with health disorders. The results showed neither significantly beneficial nor harmful results (37). A current literature review indicates no published studies on the effects of intercessory prayer on the prevention of collective violence or war. Similarly, to our knowledge, there are no published controlled studies on the effect of other types of meditation methods on public health outcomes.

### Mechanisms of group meditation in preventing collective violence

#### Population health

Drawing on the public health model of the host-agent-environment framework, Levy translated these terms for preventing collective violence to people, weapons/military, and conditions in which people live (10). Using this framework, we suggest that a traditional group meditation practice addresses the people or host component by reducing stress and increasing neurophysiological coherence and associated health conditions that may reduce collective stress and violence.

#### Population neuroscience

Population neuroscience also known as collective neuroscience, examines how cognitive processes, brain functions, and behavior interact with and mutually influence larger social environmental factors (39). In the words of Falk and colleagues, it is where *neuroscience meets population science* (40). Within this framework, group meditation programs may be viewed as a collective cognitive neuroscience stabilizing activity. These programs may synchronize individual cognitive and neurophysiological states, leading to a shared, enhanced cognitive experience in the community.

#### Distributed cognition model

As proposed by Sloman and colleagues, cognitive neuroscience processes are not confined to individual brains but are distributed across a community or potentially large group of people, thereby influencing collective behavior of the population (39).

#### Impact of group meditation on collective behavior

The coherence and peace experienced individually in meditation may be projected outward, influencing the collective cognitive neuroscience of the population. This could lead to observable changes, such as a decrease in collective violence. In the case of group meditation according to the principles described above, this shared cognitive neuroscience state may contribute to societal changes, such as a reduction in collective behavioral violence.

#### Physics principles

The principles of interconnectedness and nonlocality from quantum physics provide a theoretical basis for understanding these phenomena. In this context, actions or states in one part of a system (individual mind or consciousness) may affect distant parts of the system (collective consciousness) (41, 42).

#### Unified field of consciousness

Expanding on these concepts, Nader hypothesizes that through Traditional Medicine practices of Transcendental Meditation and the TM-Sidhi program, individuals can access a unified field of consciousness that transcends individual awareness and contributes to a collective harmonized state of neuroscience and behavior (43).

The approach presented in this perspective, rooted in cognitive and population neuroscience and models from quantum physics, offers a novel perspective on how individual cognitive practices, notably a traditional Vedic medicine meditation program, can have far-reaching impacts on public health and societal well-being.

#### Systems medicine and public health

A model of whole health that connects individual and environmental health based on advances in mind–body medicine and population health has been proposed that demonstrates the inter-relationships of these domains and the reciprocal influence between individual health and collective health (27). This paradigm is derived from traditional Vedic medicine and modern scientific evidence in a systems science, medicine and public health framework. The model is called the *Connectome of Health* (27).

### A new paradigm

Despite progress in modern public health and preventive medicine, widespread collective violence and armed conflicts continue, illustrated by the current Israel-Hamas war and other “intractable” wars. A range of measures have been recommended to prevent war but without adequate success to date (44).

The incorporation of group meditation in public health strategies for mitigating collective stress and violence, despite robust scientific validation and neuroscientific explanations, represents a paradigmatic shift as delineated by Kuhn in *The Structure of Scientific Revolutions* (45). The transition from a conventional biomedical paradigm to a biopsychosocial systems model, which incorporates the burgeoning field of population neuroscience, could disrupt prevailing perspectives. The practice of group meditation for peace represents a paradigm shift from an external locus of change to an internal one, where cultivating inner peace within individuals can lead to positive outcomes on a societal scale.

However, as Ho observes in his critique of the history of science, medicine, and public health, the relationship between evidence and theory largely depends on individual and collective worldviews for their interpretation and acceptance of new scientific findings and technologies (46).

## Conclusion

To address the critical public health need for prevention of collective stress, violence, and war, we present a framework that integrates Traditional Medicine with current scientific understanding and evidence. In this perspective, we propose that group meditation practices can effectively diminish collective stress and violence, a hypothesis supported by over 30 peer-reviewed and published field studies. This strategy is in line with population salutogenesis, which focuses on the societal factors contributing to mental distress and the escalation of collective stress and violence (47).

The evidence supporting the group practice of a traditional medicine meditation program, the Transcendental Meditation and TM-Sidhi program, suggests that this method offers more than individual benefits; it could be a vital part of a broad, evidence-based strategy for societal well-being, public health, and global peace.

## Ethics statement

Ethical approval was not required for the study involving humans in accordance with the local legislation and institutional requirements. Written informed consent to participate in this study was not required from the participants or the participants’ legal guardians/next of kin in accordance with the national legislation and the institutional requirements.

## Author contributions

RS: Conceptualization, Project administration, Resources, Supervision, Writing – original draft, Writing – review & editing. MD: Conceptualization, Formal analysis, Methodology, Resources, Writing – original draft, Writing – review & editing. GY: Investigation, Writing – original draft, Writing – review & editing. TN: Conceptualization, Resources, Writing – review & editing.
